# Vacuum Thermal Treated Ni-CeO_2_/SBA-15 Catalyst for CO_2_ Methanation

**DOI:** 10.3390/nano8100759

**Published:** 2018-09-26

**Authors:** Luhui Wang, Hui Liu, Han Ye, Rong Hu, Shuqing Yang, Guoli Tang, Kunqiang Li, Yanpeng Yang

**Affiliations:** 1 Department of Chemical Engineering, School of Petrochemical Technology and Energy Engineering, Zhejiang Ocean University, Zhoushan 316022, Zhejiang, China; Yehan18zjou@163.com (H.Y.); hurong520184@163.com (R.H.); ysqjane@163.com (S.Y.); 17805808960@163.com (G.T.); a17858802521@163.com (K.L.); m17858805428@163.com (Y.Y.); 2 School of Food and Pharmaceutical, Zhejiang Ocean University, Zhoushan 316022, Zhejiang, China; liuhui@zjou.edu.cn

**Keywords:** nickel, CeO_2_, SBA-15, vacuum thermal treatment, CO_2_ methanation

## Abstract

Ni-CeO_2_/SBA-15-V catalyst was prepared by the impregnation method with vacuum thermal treatment and used for CO_2_ methanation reaction. Compared with Ni-CeO_2_/SBA-15-air catalyst with thermal treatment in air, the reduced Ni-CeO_2_/SBA-15-V catalyst with vacuum thermal treatment exhibited higher Ni dispersion and smaller Ni particle size. In CO_2_ methanation reaction, the Ni-CeO_2_/SBA-15-V catalyst was more active and selective than the Ni-CeO_2_/SBA-15-air catalyst. The good activity and selectivity of Ni-CeO_2_/SBA-15-V catalyst should be due to highly dispersed Ni in contact with small CeO_2_ particles.

## 1. Introduction

With the increasing consumption of fossil fuel, CO_2_ released into the atmosphere has resulted in climate change and global warming, and the utilization of CO_2_ has received much attention. CO_2_ hydrogenation is an effective way to convert CO_2_ into fuels and chemicals [1,2]. CO_2_ methanation can convert CO_2_ and renewable H_2_ into methane, which is a promising process for CO_2_ conversion and the storage of renewable H_2_ [3]. 

CO_2_ methanation is an exothermic reaction, and the low reaction temperature is favorable for the high equilibrium conversion of CO_2_. Therefore, various catalysts have been developed for CO_2_ methanation at low temperature. Noble metal-based catalysts, such as Rh [4], Ru [5], and Pd [6], and non-noble Co [7,8] and Ni-based catalysts [9,10] are the most studied CO_2_ methanation catalysts. Although noble metal-based catalysts possess good activity for low-temperature CO_2_ methanation, Ni-based catalysts are preferred in industrial processes due to the low cost, and they have been applied for CO methanation [11,12] and CO_2_ methanation [13]. Ni supported on various metal oxides, such as SiO_2_ [13], Al_2_O_3_ [14], CeO_2_ [15], ZrO_2_ [16], and TiO_2_ [17], has been reported for CO_2_ methanation. Ni supported on SBA-15, which is a kind of mesoporous SiO_2_ with high specific surface area, has been reported for CO_2_ methanation [18,19]. Adding CeO_2_ to a Ni-based catalyst can enhance Ni dispersion and CO_2_ adsorption and dissociation [20]. CeO_2_-promoted Ni/SBA-15 catalyst has been used for CO_2_ methanation reaction and exhibits excellent catalytic performance [10,19].

However, it is difficult to obtain highly dispersed Ni particles on SBA-15 support by the conventional impregnation method, and Ni particles are mainly located on the SBA-15 outer surface [19]. Various methods, such as two solvents [21], surfactant assistant [22], and ammonia evaporation [23] methods have been used to improve the dispersion of Ni on SBA-15 support. Recently, vacuum thermal treatment was applied to synthesize highly dispersed Cu [24], Pt [25], and Pd [26] particles on mesoporous SBA-15. In the present work, the Ni-CeO_2_/SBA-15 catalyst was prepared by the impregnation method with vacuum thermal treatment and used for CO_2_ methanation. The vacuum thermal treated catalyst exhibited high dispersion of Ni and CeO_2_ and showed high activity and selectivity for CO_2_ methanation reaction.

## 2. Materials and Methods 

### 2.1. Synthesis of Catalysts

SBA-15 was prepared by a hydrothermal method according to the literature [27]. First, 16.4 g of P123 (EO_20_PO_70_EO_20_, Mw = 5800; Sigma Aldrich, St. Louis, MO, USA) was dissolved in 500 mL of a 2 M HCl aqueous solution. After stirring at 40 °C for 1 h, 34.0 g tetraethoxysilane, (TEOS, Sinopharm, Shanghai, China) was dropped into the solution. Then it was stirred at 40 °C for 4 h, and aged at 100 °C for 48 h in sealed Teflon bottles. SBA-15 was obtained after filtration, drying at 80 °C overnight, and calcination at 600 °C for 4 h (heating rate of 1 °C/min). 

Then, 1 g of SBA-15 was impregnated with controlled amounts of Ni(NO_3_)_2_·6H_2_O and Ce(NO_3_)_3_·6H_2_O using 5 mL of ethanol as solvent and kept at room temperature for 24 h, then it was calcinated in air or vacuum at 600 °C for 4 h with a heating rate of 1 °C/min. The resulting samples calcinated in air and vacuum are referred to as Ni-CeO_2_/SBA-15-air and Ni-CeO_2_/SBA-15-V, respectively. 

For comparison, using γ-Al_2_O_3_ (241 m^2^/g) as support, a Ni-CeO_2_/Al_2_O_3_ catalyst was prepared by impregnation method and calcinated in air as described above. 

In these catalysts, the theoretical Ni and CeO_2_ content was 10 wt % for both.

### 2.2. Characterization of Catalysts

N_2_ isotherms were measured using an Autosorb-iQ analyzer (Quantachrome Instruments, Boynton Beach, FL, USA) at −196 °C, and the pore size distribution was calculated from the desorption branch by Barrett–Joyner–Halenda (BJH) method. X-ray diffraction (XRD) patterns were measured by a DX-2700 X-ray diffractometer (Haoyuan Instrument, Dandong, China) using Cu K_α_ radiation. Transmission electron microscope (TEM) images were measured on a Tecnai G2 F20 microscope (FEI Company, Hillsboro, OR, USA). (XRD). H_2_-temperature programmed reduction (H_2_-TPR) patterns were measured on a TP-5080 adsorption instrument under a 5% H_2_/Ar flow at a rate of 10 °C/min to 900 °C. Before H_2_-TPR measurements, the samples were purged with an argon flow at 400 °C for 5 min. The outgas was monitored by a thermal conductivity detector (TCD) detector. Thermo gravimetric (TG) was carried out using an HCT-1 TG thermal analyzer (Henven Scientific Instrument, Beijing, China). The sample was heated from room temperature to 900 °C with a heating rate of 10 °C/min in air (20 mL/min).

### 2.3. Catalytic Test

The reaction was carried out in a fixed bed quartz reactor (inner diameter (ID) = 8 mm) at atmospheric pressure. The temperature of the reactor was controlled by a thermocouple inserted into the catalyst bed, and the gas flow rates were controlled by mass flow controllers. For a catalytic test, 100 mg of catalyst (60–100 mesh) diluted with 200 mg of inert SiO_2_ was used. The Ni-CeO_2_/SBA-15 catalyst was reduced to 50 mL/min of 20% H_2_/Ar stream at 450 °C for 40 min, and. After reduction, the reactor temperature was decreased to 200 °C in 20% H_2_/N_2_. Then the reactant gases (CO_2_:H_2_:Ar = 1:4:5, 100 mL/min) were introduced into the reactor. The reaction was conducted in the temperature range from 200 to 450 °C. The catalyst was kept for 1 h at each reaction temperature before the products were analyzed. The stability tests were performed at 350 and 400 °C. Before stability test, the Ni-CeO_2_/Al_2_O_3_ catalyst was reduced at 700 °C for 40 min. The exhaust and feed gas compositions were analyzed on a GC-7900 gas chromatograph (Techcomp, Kwai Chung, China) equipped with a TDX-01 column and a TCD detector. CO_2_ conversion, CH_4_ selectivity, and carbon balance were calculated using the following formulae:

$$\begin{array}{l} {\mathrm{CO}}_{2}\;\mathrm{conversion}=\frac{{\mathrm{moles}\;\mathrm{of}\;\mathrm{CO}}_{2\;\mathrm{in}}{-\mathrm{moles}\;\mathrm{of}\;\mathrm{CO}}_{2\;\mathrm{out}}}{{\mathrm{moles}\;\mathrm{of}\;\mathrm{CO}}_{2\;\mathrm{in}}}\times 100\%\\ {\mathrm{CH}}_{4}\;\mathrm{selectivity}=\frac{{\mathrm{moles}\;\mathrm{of}\;\mathrm{CH}}_{4\;\mathrm{out}}}{{\mathrm{moles}\;\mathrm{of}\;\mathrm{CO}}_{2\;\mathrm{in}}{-\mathrm{moles}\;\mathrm{of}\;\mathrm{CO}}_{2\;\mathrm{out}}}\times 100\%\\ \mathrm{Carbon}\;\mathrm{balance}=(1-\frac{{\mathrm{moles}\;\mathrm{of}\;\mathrm{CH}}_{4\;\mathrm{out}}{+\mathrm{moles}\;\mathrm{of}\;\mathrm{CO}}_{\mathrm{out}}{+\mathrm{moles}\;\mathrm{of}\;\mathrm{CO}}_{2\;\mathrm{out}}}{{\mathrm{moles}\;\mathrm{of}\;\mathrm{CO}}_{2\;\mathrm{in}}})\times 100\% \end{array}$$

In this work, carbon balance was within ±3% for all the catalytic tests, indicating negligible carbon deposition on the catalysts.

## 3. Results and Discussion

### 3.1. Characterization of Catalysts

**Figure 1:**
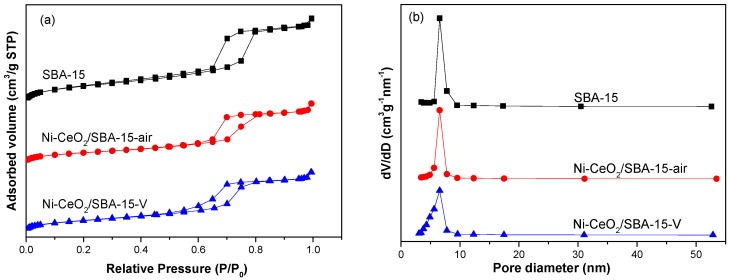
(**a**) N_2_ adsorption-desorption isotherms; (**b**) pore size distributions of SBA-15, Ni-CeO_2_/SBA-15-air, and Ni-CeO_2_/SBA-15-V.

Figure 1 shows N_2_ adsorption–desorption isotherms and pore size distributions of SBA-15, Ni-CeO_2_/SBA-15-air, and Ni-CeO_2_/SBA-15-V. All samples showed type IV isotherm with a hysteresis loop in the relative pressure 0.65–0.80, confirming the uniform mesoporous structure in these samples. As shown in Figure 1b, the pore size of all samples was 6.5 nm, indicating the addition of Ni and CeO_2_ to SBA-15 without changing the mesoporous structures. As listed in Table 1, the Brunauer-Emmett-Teller (BET) surface area and pore volume decreased after the addition of NiO and CeO_2_ into SBA-15.

**Figure 2:**
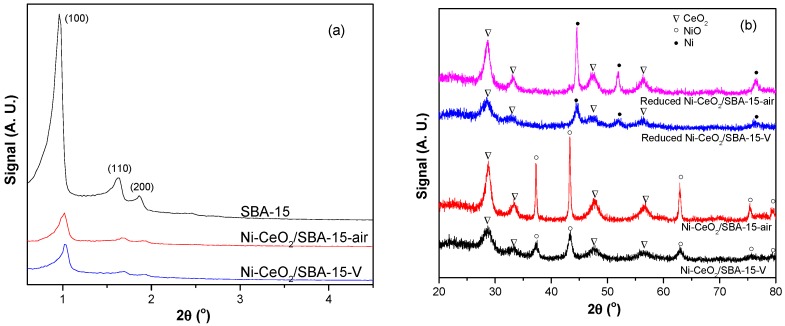
(**a**) Small-angle and (**b**) wide-angle X-ray diffraction (XRD) patterns of SBA-15, Ni-CeO_2_/SBA-15-air, and Ni-CeO_2_/SBA-15-V catalysts.

Figure 2 shows the XRD patterns of the catalysts. The reduced catalysts were reduced in a 20% H_2_/Ar stream (50 mL/min) for 40 min at 450 °C. As shown in Figure 2a, all the small-angle XRD patterns showed three typical peaks of mesoporous SBA-15 [27]. The result demonstrates that the mesoporous structure of SBA-15 was maintained after loading NiO and CeO_2_. Figure 2b shows the wide-angle XRD patterns of fresh and reduced catalysts. Compared with Ni-CeO_2_/SBA-15-air, Ni-CeO_2_/SBA-15-V exhibited obviously wider reflection peaks of NiO and CeO_2_, indicating that vacuum treatment can improve the dispersion of NiO and CeO_2_. After reduction, the reflection peaks of Ni in Ni-CeO_2_/SBA-15-V were also wider than those in Ni-CeO_2_/SBA-15-air, confirming that the Ni particle was smaller in the Ni-CeO_2_/SBA-15-V catalyst after reduction. As listed in Table 2, the crystal sizes of Ni and CeO_2_ in reduced Ni-CeO_2_/SBA-15-V were 8.5 and 4.2 nm, which were obviously smaller than those in reduced Ni-CeO_2_/SBA-15-air. The XRD results indicate that vacuum thermal treatment can improve Ni and CeO_2_ dispersion on SBA-15 support.

**Figure 3:**
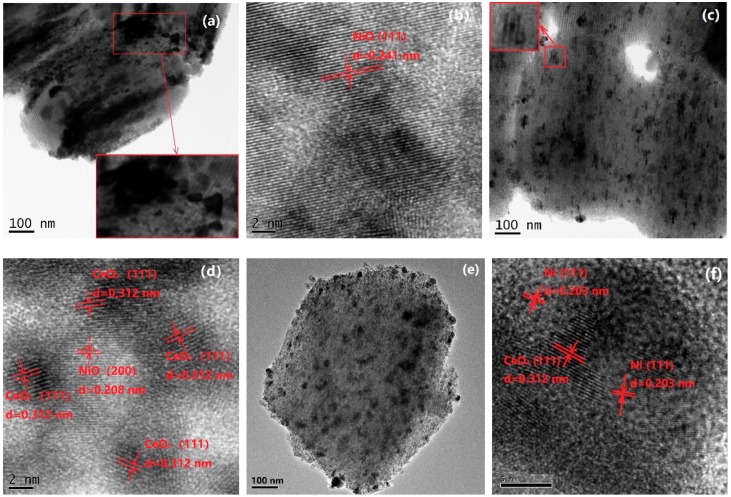
Transmission electron microscope (TEM) images of fresh and reduced catalysts: (**a**,**b**) fresh Ni-CeO_2_/SBA-15-air, (**c**,**d**) fresh Ni-CeO_2_/SBA-15-V, and (**e**,**f**) reduced Ni-CeO_2_/SBA-15-V.

Figure 3 shows TEM images of the catalysts. For all catalysts, a well-ordered mesoporous structure could be observed. For fresh Ni-CeO_2_/SBA-15-air catalyst, most particles were 20–100 nm in size, which were larger than the pore size of SBA-15 and dispersed on the outside surface of SBA-15 (Figure 3a), and NiO particles larger than 20 nm could be clearly observed in Figure 3b. On the contrary, for fresh Ni-CeO_2_/SBA-15-V (Figure 3c), lots of particles were aligned within the mesoporous channels of SBA-15, indicating this part of particles located in the pore channels. For fresh Ni-CeO_2_/SBA-15-V, small particles were dispersed on the outside surface or in the nanochannel of SBA-15. NiO particles smaller than 10 nm in contact with CeO_2_ particles were observed (Figure 3d). TEM images of reduced Ni-CeO_2_/SBA-15-V catalyst are shown in Figure 3e,f. Metal Ni particles were found to be in contact with CeO_2_ particles, as shown in Figure 3f.

**Figure 4:**
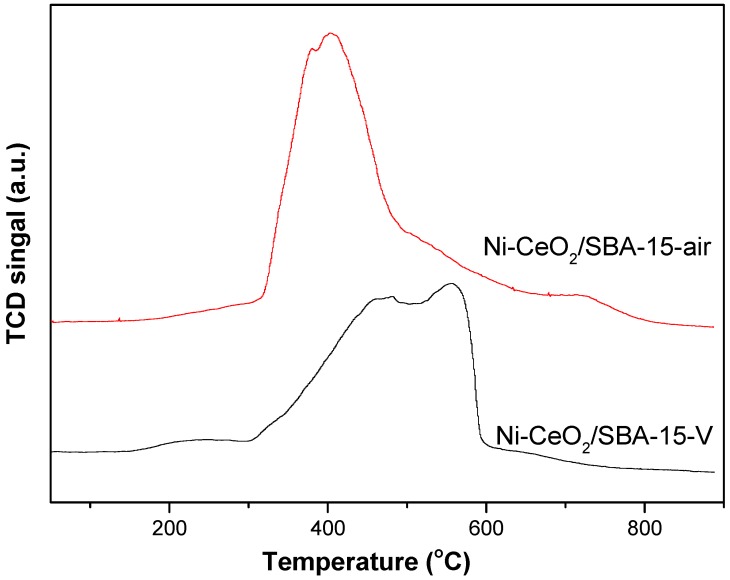
H_2_-temperature programmed reduction (H_2_-TPR) profile of Ni-CeO_2_/SBA-15-air and Ni-CeO_2_/SBA-15-V catalysts.

Figure 4 shows H_2_-TPR profiles of the catalysts. For Ni-CeO_2_/SBA-15-air, the sharp peak located at around 420 °C was attributed to the reduction of large bulk NiO species with no or very little interaction with SiO_2_ support [19]. For Ni-CeO_2_/SBA-15-V, a reduction peak between 300 °C and 600 °C was observed. A reduction peak beyond 500 °C corresponds to the reduction of small NiO particles strongly interacting with SiO_2_ support [28,29,30,31,32,33]. The result indicates that there is a strong interaction between Ni and SiO_2_ in Ni-CeO_2_/SBA-15-V catalyst.

### 3.2. Catalytic Performance

**Figure 5:**
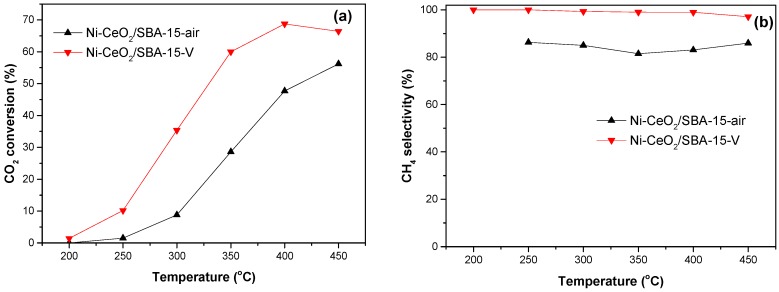
(**a**) CO_2_ conversion and (**b**) CH_4_ selectivity of Ni-CeO_2_/SBA-15-air and Ni-CeO_2_/SBA-15-V catalysts in methanation reaction.

The CO_2_ conversion and CH_4_ selectivity of the catalysts for CO_2_ methanation reaction are shown in Figure 5. Compared with the Ni-CeO_2_/SBA-15-air catalyst, the Ni-CeO_2_/SBA-15-V catalyst exhibited obviously higher CO_2_ conversion and CH_4_ selectivity. For the Ni-CeO_2_/SBA-15-V catalyst, CO_2_ conversion and CH_4_ selectivity at 400 °C were 68.8% and 99.0%, respectively. CH_4_ selectivity of the Ni-CeO_2_/SBA-15-V catalyst decreased to 97.1% at 450 °C. This is because reverse water gas shift reaction (CO_2_ + H_2_ → CO + H_2_O) is an endothermic reaction, and high temperatures are more conducive to producing CO and decreasing CH_4_ selectivity. The result shows that the Ni-CeO_2_/SBA-15-V catalyst was active and selective for the CO_2_ methanation reaction. It should be noted that the Ni-CeO_2_/SBA-15-air catalyst produced a large amount of CO even at low temperature. This may be due to that the large Ni particle in the Ni-CeO_2_/SBA-15-air catalyst was active for both CO_2_ methanation and reverse water–gas shift reaction, while small Ni particle in contact with CeO_2_ in Ni-CeO_2_/SBA-15-V catalyst was more selective for CO_2_ methanation.

**Figure 6:**
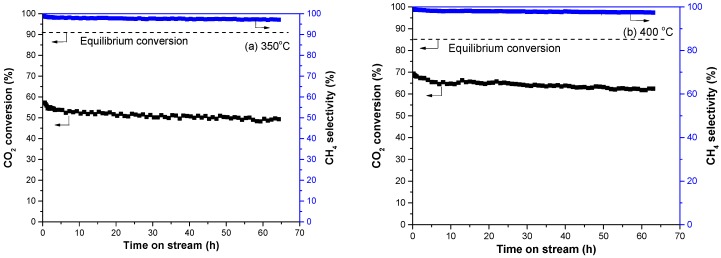
Stability of the Ni-CeO_2_/SBA-15-V catalyst in methanation reaction at (**a**) 350 °C and (**b**) 400 °C.

Figure 6 shows the stability test of Ni-CeO_2_/SBA-15-V catalyst at 350 °C and 400 °C. The equilibrium conversions are also shown in Figure 6. The CO_2_ conversions of Ni-CeO_2_/SBA-15-V catalyst had a little decrease in initial 10 h on stream, then the catalyst exhibited stable catalytic performance for 50 h at 350 °C and 400 °C. After reaction for 60 h, the CO_2_ conversion at 350 °C and 400 °C stayed at around 49.5% and 62.5%, respectively. It is worth noticing that, during the stability test at 400 °C, CH_4_ selectivity achieved over the Ni-CeO_2_/SBA-15-air catalyst remained almost constant after 10 h on stream approaching values higher than 97%.

Among Ni-based methanation catalysts, Ni/Al_2_O_3_ catalyst is the most widely investigated and used due to its low costs and availability. Table 3 shows the CO_2_ conversion and selectivity of reported Ni/Al_2_O_3_ catalyst at 250 and 300 °C. Compared with the Ni/Al_2_O_3_ catalyst, the CO_2_ conversion of Ni-CeO_2_/SBA-15-V was obviously higher.

**Figure 7:**
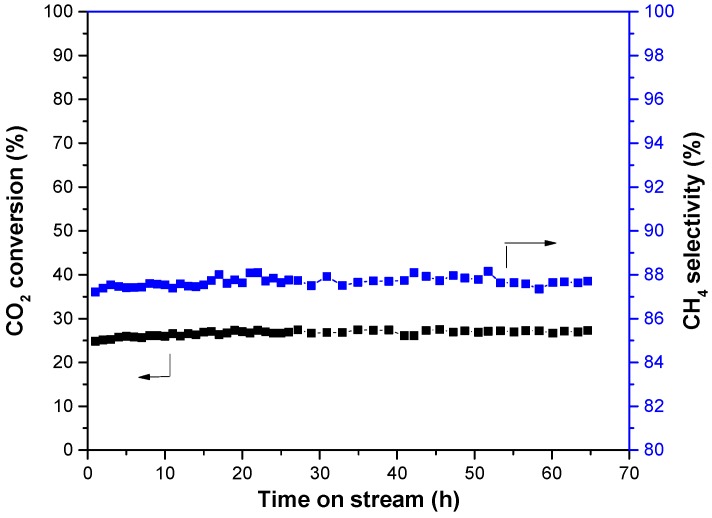
Stability of Ni-CeO_2_/Al_2_O_3_ catalysts in methanation reaction at 350 °C.

For comparison with Ni-CeO_2_/SBA-15-V catalyst, the stability of Ni-CeO_2_/Al_2_O_3_ catalyst prepared with a conventional impregnation method was tested at 350 °C. As shown in Figure 7, the CO_2_ conversion and CH_4_ selectivity of Ni-CeO_2_/Al_2_O_3_ catalyst were obviously lower than that of Ni-CeO_2_/SBA-15-V catalyst.

**Figure 8:**
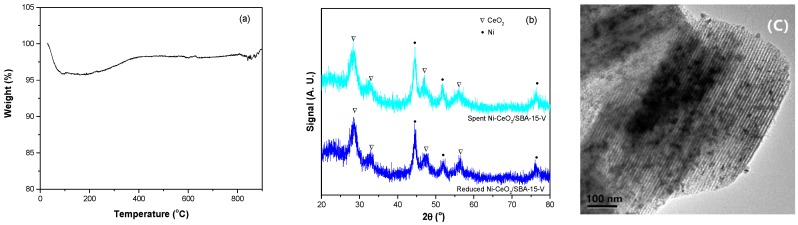
(**a**) Thermo gravimetric (TG) curve, (**b**) XRD pattern, and (**c**) TEM image of the spent Ni-CeO_2_/SBA-15-V catalyst after stability test at 400 °C.

Carbon deposition and sintering of metal particle are the main reasons for the catalyst deactivation during CO_2_ methanation [10,35,36]. The spent Ni-CeO_2_/SBA-15-V catalyst after stability test at 400 °C was characterized by TG, XRD, and TEM. TG curve was shown in Figure 8a. There was a weight decrease below 100 °C and a weight increase in the temperature region of 180–400 °C. The weight decrease should be due to the desorption of adsorbed water and the weight increase due to the oxidation metal Ni to NiO. There was no obvious weight loss above 100 °C, indicating no obviously carbon deposition was formed on the spent Ni-CeO_2_/SBA-15-V catalyst. XRD patterns of reduced and spent Ni-CeO_2_/SBA-15-V catalysts were shown in Figure 8b. Determined by Scherrer’s equation from the (111) plane of Ni in XRD patterns, Ni crystal sizes were 8.5 and 9.1 nm for reduced and spent Ni-CeO_2_/SBA-15-V catalysts, respectively. XRD results indicate that the Ni particle size was slightly increased during the stability test. As shown in Figure 8c, TEM image also showed that no obviously sintering of Ni particle occurred. The TG, XRD, and TEM results indicate the slight decrease of activity and selectivity during stability test was due to the slight increase of Ni particle size.

The present work clearly demonstrates that the Ni-CeO_2_/SBA-15-V catalyst possessed superior activity and selectivity and exhibited excellent stability during the CO_2_ methanation reaction. The XRD and TEM results prove that the vacuum-thermal treated Ni-CeO_2_/SBA-15-V catalyst had higher Ni dispersion and smaller Ni particle size than the Ni-CeO_2_/SBA-15-air catalyst thermal treated in air. Based on the characterization and catalyst performance results, it can be concluded that good activity and selectivity of Ni-CeO_2_/SBA-15-V catalyst can be attributed to the small Ni particle size in contact with CeO_2_.

## 4. Conclusions

The effects of vacuum thermal treatment on Ni-CeO_2_/SBA-15 catalysts were investigated in this paper. Compared with thermal treatment in air, vacuum thermal treatment can improve the dispersion of Ni and CeO_2_ in the Ni-CeO_2_/SBA-15 catalyst. The Ni-CeO_2_/SBA-15-V catalyst with vacuum thermal treatment exhibited better activity and selectivity than the Ni-CeO_2_/SBA-15-air catalyst thermal treated in air. The excellent catalytic performance of the Ni-CeO_2_/SBA-15-V catalyst was mainly attributed to higher Ni and CeO_2_ dispersion.

## Figures and Tables

**Table 1:** Textural properties of SBA-15, Ni-CeO_2_/SBA-15-air, and Ni-CeO_2_/SBA-15-V.

Samples	S_BET_ (m^2^/g)	Pore Volume (cm^3^/g)	Average Pore Diameter (nm)
SBA-15	690	1.12	6.56
Ni-CeO_2_/SBA-15-air	433	0.83	6.54
Ni-CeO_2_/SBA-15-V	492	0.83	6.56

**Table 2:** Physical properties of catalysts.

Catalysts	CeO_2_ Crystal Size (nm) ^a^	NiO Crystal Size (nm) ^b^	Ni Crystal Size (nm) ^c^	Ni Dispersion (%) ^d^
Ni-CeO_2_/SBA-15-air (fresh)	7.3	42.1	-	-
Ni-CeO_2_/SBA-15-air (reduced)	7.1	-	26.1	3.7
Ni-CeO_2_/SBA-15-V (fresh)	4.6	10.8	-	-
Ni-CeO_2_/SBA-15-V (reduced)	4.2	-	8.5	11.4

^a^ Determined by Scherrer’s equation from the (111) plane of CeO_2_ in X-ray diffraction (XRD) patterns; ^b^ determined by Scherrer’s equation from the (200) plane of NiO in XRD patterns; ^c^ determined by Scherrer’s equation from the (111) plane of Ni in XRD patterns; ^d^ calculated as (97.1 nm)/(particle size of Ni(nm)).

**Table 3:** CO_2_ conversion and CH_4_ selectivity of Ni-CeO_2_/SBA-15-V and reported Ni/Al_2_O_3_ catalyst.

Catalysts	Ni Content (wt %)	T (°C)	CO_2_ Conversion (%)	CH_4_ Selectivity (%)	Ref.
Ni-CeO_2_/SBA-15-V	10%	250	10.2	100	This work ^a^
		300	35.4	99.3	This work ^a^
Ni/Al_2_O_3_	14%	250	1	100	[34] ^b^
		300	6	100	[34] ^b^

^a^ Weight hourly space velocity(WHSV) = 60,000 mL g^−1^ h^−1^, CO_2_/H_2_/Ar = 1/4/5; ^b^ Gas hourly space velocity (GHSV) = 52,300 h^−1^, CO_2_/H_2_ = 1/5.
